# Mr. Ring, on Vaccination

**Published:** 1804-01-01

**Authors:** John Ring

**Affiliations:** New Street, Hanover Square


### *To the Editors of the Medical and Phyfical Journal.*

Gentlemen,

THE following extract of a letter from Dr. Marshall, dated Lymington, 6 Dec. 1803, fully confirms an opinion which I advanced in the last Number of your Publication; and, from the importance of the subject to which it relates, I flatter myself you will insert it the first opportunity.

"Since my arrival here, I have been busy with the Cow-pox, having inoculated upwards of five hundred, principally of the poor, who have not had the Small-pox amongst them for the last twelve years.

"In this month's Medical Journal, I observe a letter from Mr. Weston, of Jamaica, to you, is inserted; in which he says, he is 'obliged to acknowledge the lamentable fact, that in a temperature of 90, the vaccine matter loses its activity.'

"This, to me, appears a very extraordinary acknowledgment; for during the time that I was at Gibraltar, in August 1800, the temperature was never so low as 90, and frequently 96: during which time I never observed any particular difficulty in communicating infection more than in England; nor can I conceive any probable cause for its failure in Jamaica.

"Mr. Weston also says, he has 'ascertained, beyond cavil, that the negro habit is susceptible of the disease." Upon this point I should have supposed there could not be be two opinions. One of the first persons vaccinated on board the Endymion, in 1800, with matter furnished by you, was a negro; and his arm furnished the virus for our future inoculations.

"From my original memorandum, now before me, I find your observation upon the rapid progress of the disease in Negroes just; since the arm of this man yielded matter for inoculation on the third day.

"I am well convinced, that if proper precautions be taken, vaccine virus will not become effete in any climate. At Palermo, I used matter taken in Malta eighteen days before, with perfect success. The same matter, used at Naples three months after, still retained its virtue, though the temperature was frequently upwards of 100.

"Virus taken upon ivory or quill, wrapped in paper, and kept in a drawer, is, I believe, capable of being preserved as long as can be necessary in any climate."

To this valuable communication from Dr. Marshall, I beg leave to subjoin a few miscellaneous observations.

In my Treatise on the Cow-pox, p. 799, I stated, that Dr. Jenner had been requested by the Physicians and Surgeons who had presented him with a gold medal, to sit for his picture to Northcote. This was a mistake. It was the Medical Society of Plymouth, consisting exclusively of the resident physicians of Plymouth, Dock, and Storehouse, who gave this additional testimony of the high esteem in which they hold the discovery of Dr. Jenner.

The last, but not the least honour conferred on Dr. Jenner, is a Latin Ode on the subject of Vaccination, written by the celebrated Anstey, author of the New Bath Guide, and other excellent poems.

I have lately met with a case, which is, perhaps, unparalleled in the annals of inoculation; in which the sign of infection did not appear till the forty-sixth day. A very small, but genuine, vaccine vesicle then took place. The child has since been inoculated repeatedly with the most active matter, but to no purpose.

The subject in whom this remarkable circumstance occurred, is a child of Mr. Dust, a gun-maker, at Chelsea. There is reason to believe, that the effect of the vaccine virus was retarded by the counter-stimulus of a very large tetter on the shoulder of the opposite arm. This tetter was above three inches and a half in length, one inch and a half in breadth, and of long standing.

About

About a week after the insertion of vaccine virus, the tetter was more inflamed; but from that time it began to mend, and in a few weeks totally disappeared.

*New Street, Hanover Square,*
*December* 14, 1803.

I am, &c.
JOHN RING.

